# Herd immunity at what price? Using auctions to estimate what university students must be paid to get the flu vaccine

**DOI:** 10.1016/j.pmedr.2021.101466

**Published:** 2021-06-23

**Authors:** Nick Clark, Jay R. Corrigan, Matthew C. Rousu

**Affiliations:** a Susquehanna University, United States; b Kenyon College, United States

## Abstract

There is significant resistance to vaccinations. Fewer than half of adults get a flu shot in the United States in a typical year, and a large minority of Americans say they will not get vaccinated against COVID-19. This resistance to vaccines creates challenges for both public health and the economy. The academic literature needs to consider potential policy solutions that might increase vaccination rates. In this study, we use experimental auctions to estimate how much university students need to be paid in exchange for agreeing to get a flu shot. These were real auctions where winners received compensation to get the flu shot. As found in prior research, the perceived stakes of such auctions incentivize participants to estimate the price at which they would engage in the auctioned behavior – in this instance, receiving a flu shot. We find that 50% require less than $1, and an additional 30% would get vaccinated for a payment of $20 or less. We also use a tobit regression to estimate bids as a function of participants’ demographic characteristics. If low levels of compensation increase vaccination rates, this has significant public health implications. The government may be able to achieve higher vaccination rates at a relatively low cost, particularly in comparison with the economic harms caused by illness. This study demonstrates that experimental auctions may be useful for estimating how much a larger, more representative sample would need to be paid in exchange for agreeing to receive flu or COVID-19 vaccinations.

## 1 Introduction

The rapid spread of SARS-CoV-2, the virus that causes COVID-19, has upended the global economy, transformed the nature of work for many professions, disrupted social and political relationships, and brought into sharp relief the reluctance of many individuals to sacrifice even marginal personal freedoms for public health concerns. Aversion to following public health recommendations is not new for the American public. Many Americans are hesitant to receive vaccines; indeed, fewer than half of American adults get the flu shot annually despite the Centers for Disease Control and Prevention’s recommendation that all Americans over the age of 6 months get vaccinated (Centers for Disease Control and Prevention., 2019). While the flu vaccine is not 100% effective, experts agree it is one of the best ways to help prevent the flu (Centers for Disease Control and Prevention., 2020). The public health and economic consequences of the seasonal flu are dire. The CDC estimates that 38 million Americans contracted the flu in the 2019–20 season, resulting in 400,000 hospitalizations and 22,000 deaths. In addition to the loss of life, the flu leads to $10 billion in annual medical costs and an estimated $87 billion in lost work (Dobson, 2007).

Vaccination rates are frustratingly low among university students. Studies consistently find that university students are less likely to get vaccinated against the flu than adults in general, with estimates of vaccination rates ranging from 15% to 30% (Nichol et al., 2008, Poehling et al., 2012, Ravert et al., 2012). This is especially concerning given that many students live in close proximity to one another during the academic year, then travel home over breaks to visit family, some of whom may be elderly or very young (Bednarczyk et al., 2015). And while young adults have traditionally been thought of as being at lower risk from the flu, the strains circulating in 2009–10 (Centers for Disease Control and Prevention (CDC), 2010) and 2013–14(Centers for Disease Control and Prevention (CDC), 2013) hit young adults especially hard.

Why do university students choose not to get vaccinated? Some have concerns about the safety of vaccines, others might not be able to find the time to get vaccinated, and still others might not think they need a vaccination given that they are in good health (Bednarczyk et al., 2015). It may be possible to persuade some of these hesitant students to get vaccinated by paying them to do so. State and federal governments often use fiscal and tax policies to promote certain behavioral choices among the public, including incentives for pursing a college education, buying a home, and installing renewable technologies, or by creating financial disincentives for remaining uninsured. The government might use similar incentives to persuade people to receive an annual flu vaccine. Such a policy could increase the number of vaccinated individuals, potentially saving thousands of lives and billions of dollars annually.

Bronchetti et al. (2015) offer university students $30 to receive a flu vaccination. The authors find that students offered this incentive were twice as likely to get vaccinated as those in a control group. In this paper, we present the results of a study using experimental auctions to estimate the compensation that university students require in order to receive a flu shot. This allows us to precisely estimate the payment individual students require to get vaccinated and to estimate the incentive required to meet a herd immunity threshold where a large enough percentage of the population is immune, making person-to-person spread more difficult (e.g., 70% immunization) (D’Souza and Dowdy, 2021).

We used real auctions where the winners received compensation to get the flu shot. As the auction participants faced real and immediate financial consequences, they had a stronger incentive to take the exercise seriously and to answer truthfully than if the questions had been hypothetical. Experimental auctions have been used to estimate how graphic warning labels affect smokers’ demand for cigarettes (Thrasher et al., 2011), to determine how teens’ demand for e-cigarettes responds to an increase in price (Quisenberry et al., 2020), and, most relevant to the current study, how much adult smokers must be paid to quit smoking for one week (Quisenberry et al., 2020).

The methods and results presented here not only speak to flu vaccinations on university campuses, but may also inform potential responses to the COVID-19 vaccines. Thirty percent of respondents in a recent Pew survey indicated that they probably or definitely would not take COVID-19 vaccine (Carly and Tyson, 2021). Some of these individuals will assuredly refuse a vaccine under any circumstances, but policymakers may be able to incentivize enough of these anti-vaccination individuals to achieve a greater degree of uptake.

## 2 Methods

The Institutional Review Board at Susquehanna University approved this study. Sixty-six undergraduates at Susquehanna University – a small liberal arts university in central Pennsylvania – participated in the auction in five groups of 12 to 15 at a time. Participants were recruited via campus-wide email and received $20 for taking part in this 30-minute study in October 2020, immediately after the completion of the university’s vaccination drive. Participants had not received a flu vaccination that year.

The auction experiment had four steps.

Step 1: Instructions

The experimenter read instructions aloud while participants followed along in written instructions that closely followed those in Corrigan et al. (2018), which were adapted from Plott et al. (2005). Participants learned they would be bidding in a second-price Vickrey (1961) auction where the lowest bidder would receive the second-lowest bid in exchange for selling something they owned or completing a task. For example, if the lowest bid in a group was $1 and the second-lowest bid was $3, the person who submitted the $1 bid would receive $3 in exchange for being vaccinated. The Vickrey auction is demand revealing. Because a participant’s bid does not affect the price they receive if they win the auction, the participant can do no better than to submit a bid equal to what they would truly be willing to accept. The instructions emphasized this using logic and numerical examples.

Step 2: Practice auction

Each participant received a pen, which the experimenter explained was theirs to keep at the end of the study. Participants then bid to sell their pen in a second-price auction. After a experimenter collected these bids, he announced the lowest bidder’s ID number and explained that that participant would receive the second-lowest bid in exchange for their pen at the end of the experiment. The purposes of this practice auction were to familiarize participants with the auction framework and to show this was a real auction (i.e., that the lowest bidder would be paid the second-lowest bid in exchange for something).

Step 3: Vaccination auction

Participants bid in an auction to receive a flu vaccination. The experimenter explained that the lowest bidder would receive a flu shot. The lowest bidder in each group was given a CVS gift card which covered the cost of the flu shot. All five winning participants verified they received the flu shot, at which point they received compensation equal to the second-lowest bid.

Step 4: Survey

After the experimenter collected the bids but before he announced the results of the auction, participants completed a survey with question about whether they had received a flu shot the previous year, whether they planned to get one this year, their attitudes toward vaccines in general, their attitudes toward COVID-19, who they planned vote for in the 2020 presidential election, and basic demographic information. After participants submitted their surveys, the researcher announced the winner of the vaccination auction, dismissed participants individually, and the lowest bidder received the CVS gift card mentioned above.

## 3 Results

**Fig. 1:**
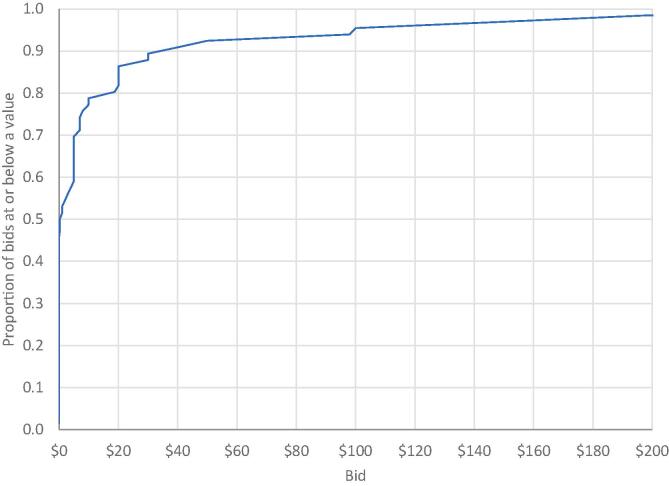
Cumulative distribution of vaccination auction bids.

Table 1 presents summary statistics from the auctions and the survey. Fig. 1 presents the cumulative distribution of vaccination bids. Note that 70% of our participants would be willing to receive a flu vaccination in exchange for $5 or less. Table 2 presents the results from a tobit regression accounting for bids being censored from below at $0. These results exclude the responses of one participant who bid $100,000 in the vaccination auction. Participants who said they planned to get a COVID-19 vaccination as soon as it becomes available required $32 less in compensation to receive a flu shot. This result is statistically significant at the 0.01 level. Participants who said they plan to vote for Joe Biden in the 2020 presidential election required $35 less in compensation to receive a flu shot. This result is statistically significant at the 0.05 level. No other coefficient estimates were statistically significantly different from zero.

**Table 1:** Summary statistics from auctions and survey (*N* = 66).

Variable	Mean
Practice bid indicating compensation required to sell pen	$2.45 [$1.75]^a^ ($3.22)^b^
Vaccination bid indicating compensation required to receive flu shot	$13.78^c^ [$0.25] ($34.79)
Did you get a flu shot last year? (1 if yes)	0.42
Were you planning to get a flu shot? (1 if yes)	0.61
I'm afraid of needles. (1 agree or strongly agree)	0.27
In general, I think vaccines are safe and effective. (1 if agree or strongly agree)	0.91
If a COVID-19 vaccine becomes available, I'll get vaccinated right away. (1 if agree or strongly agree)	0.55
Pennsylvania's state-wide mask order is a good idea. (1 if agree or strongly agree)	0.92
COVID-19 is a public health emergency. (1 if agree or strongly agree)	0.97
I have confidence in what state and local officials say about COVID-19. (1 if agree or strongly agree)	0.56
I have confidence in what federal officials say about COVID-19. (1 if agree or strongly agree)	0.36
I stay well informed about COVID-19. (1 if agree or strongly agree)	0.82
What do you think about general lockdowns as a response to COVID-19? (1 if not strict enough)	0.56
How will you vote in the upcoming election? (1 if Biden)	0.68
What is your gender? (1 if female)	0.67

a Median in brackets.

b Standard deviation in parentheses.

c Excludes one bid of $100,000. Including this bid, mean, median, and standard deviation are $1,528.72, $0.63, and $12,307.50.

**Table 2:** Tobit regression results where vaccination bid is the dependent variable (*N* = 65).

Variable	Coefficient estimate	Standard error
Constant	−15.08	47.02
Did you get a flu shot last year? (1 if yes)	−7.94	16.20
Were you planning to get a flu shot? (1 if yes)	−21.81	15.41
I'm afraid of needles. (1 agree or strongly agree)	−14.65	12.34
In general, I think vaccines are safe and effective. (1 if agree or strongly agree)	11.82	19.85
If a COVID-19 vaccine becomes available, I'll get vaccinated right away. (1 if agree or strongly agree)	−32.08	11.91
Pennsylvania's state-wide mask order is a good idea. (1 if agree or strongly agree)	42.17	21.08
COVID-19 is a public health emergency. (1 if agree or strongly agree)	20.87	35.11
I have confidence in what state and local officials say about COVID-19. (1 if agree or strongly agree)	−2.22	15.03
I have confidence in what federal officials say about COVID-19. (1 if agree or strongly agree)	−5.22	14.92
I stay well informed about COVID-19. (1 if agree or strongly agree)	24.62	15.93
What do you think about general lockdowns as a response to COVID-19? (1 if not strict enough)	−3.14	13.40
How will you vote in the upcoming election? (1 if Biden)	−35.07	13.80
What is your gender? (1 if female)	−15.19	12.16

## 4 Discussion

Most participants required little if any compensation to receive a flu shot. Half of participants required less than $1 in compensation in exchange for being vaccinated, 70% required no more than $5, and 85% required no more than $20. This is particularly noteworthy given the timing of the study. Because our auctions took place after a campus-wide vaccination drive, and our participants had opted not get vaccinated, they were not among the students most eager to get vaccinated. This suggests university or public health officials looking to achieve herd immunity could increase the vaccination rate among university students at low cost by offering students modest payments or other types of incentives.

The effects attitudes toward a COVID-19 vaccine and political affiliation have on participants’ bids are not only statistically significant, they are also large. The $32 and $35 effects are roughly equal to the $35 standard deviation of vaccination bids reported in Table 1. Biden supporters were more willing to take basic public health steps like getting a flu shot. These findings confirm other research that left-leaning individuals may be less hesitant to question public health mandates, particularly in response to the COVID-19 pandemic (Taylor-Clark et al., 2005, Morain and Mello, 2013).

At the same time, these results speak to the strength of resistance to vaccines. Often, the anti-vaccination movement presents itself (and is in turn portrayed) as fundamentally and unwaveringly opposed to such vaccine mandates. Their opposition to vaccines seems to be absolute. While this is undoubtedly true for some individuals, all participants in our study were willing to accept a vaccine for a certain price, and for the overwhelming majority this was less than $100. In other words, most pockets of opposition to vaccines are persuadable on this topic.

Perhaps the more important of these findings is that attitudes toward the COVID-19 and flu vaccines are correlated. While polling at the time our study was conducted suggested that only about half of Americans would try to get vaccinated once a COVID-19 vaccine becomes available (SSRS, 2020), our finding that attitudes toward COVID-19 and flu vaccines are correlated suggests public health officials may want to focus their efforts on promoting a COVID-19 vaccine in areas where people have traditionally been least likely to receive a flu shot, such as states in the Northern Great Plains and the Southeast (Centers for Disease Control and Prevention, 2019). While some might balk at the idea of incentivizing vaccines through monetary payments, incentives may be ethically preferable to mandatory vaccination (Savulescu, 2021), and an incentive policy may save thousands of lives and reduce the need for further economy-wide lockdowns.

In the future, researchers should extend this auction methodology to a larger, more demographically representative sample, to other types of university settings (e.g., large public universities or community colleges), and should consider focusing on populations that have historically been most hesitant to get vaccinated. Now that COVID-19 vaccines are available, this auction methodology could be used to understand if compensation could increase the vaccination rate.

## CRediT authorship contribution statement

**Nick Clark:** Conceptualization, Writing - review & editing, Funding acquisition. **Jay R. Corrigan:** Conceptualization, Methodology, Formal analysis, Writing - review & editing. **Matthew C. Rousu:** Conceptualization, Methodology, Writing - original draft, Project administration, Funding acquisition.

## Declaration of Competing Interest

The authors declare that they have no known competing financial interests or personal relationships that could have appeared to influence the work reported in this paper.
